# Early Experience With Robotic-Assisted Radical Cystectomy: A Six-Case Series From a UK Centre

**DOI:** 10.7759/cureus.94878

**Published:** 2025-10-18

**Authors:** Jonathan McAdam, Andrew McAdam, Hafs Elhag, Ramprakash Beekharry

**Affiliations:** 1 General Surgery, Belfast Health and Social Care Trust, Belfast, GBR; 2 Urology, Belfast Health and Social Care Trust, Belfast, GBR

**Keywords:** ileal conduit, implementation of robotic-assisted radical cystectomy, robotic-assisted radical cystectomy, ureteral anastamosis, urologic oncology

## Abstract

Background: Robotic-assisted radical cystectomy (RARC) is increasingly adopted as a minimally invasive alternative to open radical cystectomy for muscle-invasive and high-risk non-muscle-invasive bladder cancer. This study presents early outcomes from the first six robotic cystectomies performed at a UK tertiary centre.

Methods: A retrospective review was performed of the first six patients who underwent RARC between May and July 2025. Data collected included patient demographics, operative details, complications (graded by Clavien-Dindo), pathology, and follow-up outcomes.

Results: The median age was 65.5 years (range: 54-70), with four males and two females. The median body mass index (BMI) was 29.1. The median total operative time was 466.5 minutes (range: 429-511), with estimated blood loss ranging from 200 to 900 mL. No patients required intraoperative transfusion or conversion to open surgery. Two patients developed postoperative ileus and one experienced stoma retraction; all were managed conservatively. There was no 30- or 90-day mortality. Pathology demonstrated stages ranging from Ta to T2a, with negative surgical margins in five cases, and one result awaited at the time of reporting. At an early median follow-up period, all patients were alive without evidence of recurrence.

Conclusions: RARC was safely introduced at our centre, with perioperative outcomes comparable to early series internationally. Ongoing data collection and longer follow-up are required to assess oncological durability and functional outcomes.

## Introduction

Bladder cancer represents a significant global health burden, ranking as the tenth most common cancer worldwide and accounting for considerable morbidity and mortality [1]. In the United Kingdom, bladder cancer is the 11th most common cancer, with approximately 10,300 new cases annually [2]. Muscle-invasive bladder cancer (MIBC) carries a particularly poor prognosis if left untreated, with five-year survival rates dropping below 50% [3]. Radical cystectomy (RC) with urinary diversion remains the gold standard for curative management of MIBC and for selected cases of high-risk non-muscle-invasive bladder cancer (NMIBC) [4, 5].

Despite its role as the cornerstone of curative therapy, RC is associated with high perioperative morbidity, with complication rates reported in up to 60% of cases [6]. Open radical cystectomy (ORC), traditionally performed via a midline laparotomy, often requires extended hospital stays and is linked with significant blood loss, transfusion requirements, and prolonged recovery [7]. The development of minimally invasive techniques, first with laparoscopy and subsequently with robotic-assisted approaches, has aimed to reduce this morbidity while maintaining oncological equivalence.

Robotic-assisted radical cystectomy (RARC) has gained traction worldwide, with evidence suggesting benefits in perioperative recovery, including reduced blood loss, earlier return of bowel function, and shorter length of stay, albeit with longer operative times [8, 9]. Importantly, randomised controlled trials such as the RAZOR trial have demonstrated the non-inferiority of RARC compared with ORC in terms of progression-free survival [9]. The European Association of Urology (EAU) and NICE guidelines now acknowledge RARC as an acceptable alternative to ORC in appropriately selected patients [10-12].

The successful adoption of RARC depends not only on surgical expertise but also on institutional readiness, including access to robotic platforms, trained teams, and enhanced recovery pathways [13]. Learning curve studies suggest that proficiency in RARC requires 30 to 50 cases, with further improvement in efficiency and complication rates seen with higher volumes [14, 15]. Early case series from centres worldwide have demonstrated feasibility and safety, but the generalisability of outcomes depends on reporting from new centres adopting this technology.

We present the outcomes of the first six robotic cystectomies performed at a UK tertiary centre, providing insights into perioperative safety, pathology results, and short-term follow-up. This series contributes to the growing body of evidence supporting the safe introduction of RARC into routine practice, particularly within the NHS framework.

## Materials and methods

A retrospective analysis was performed of the first six consecutive patients who underwent RARC at our institution between May and July 2025. All cases were discussed in a multidisciplinary team (MDT) meeting and were deemed suitable for RARC based on tumour stage, comorbidities, and patient preference. Inclusion criteria included patients with MIBC or high-risk NMIBC confirmed on histopathology following transurethral resection of bladder tumour (TURBT) undergoing planned radical cystectomy. Patients undergoing palliative procedures or those with evidence of metastatic disease were excluded.

Operative surgeon

The primary surgeon in these cases was an experienced robotic and open surgeon with a high-volume robotic prostatectomy and open cystectomy practice. In preparation for launching the robotic cystectomy service at our centre, they completed training at two large-volume centres in England and Ireland to observe and learn the procedure, followed by attendance at a robotic cystectomy wet skills lab in Europe. For the first two cystectomies in this series, there was a proctor in attendance, followed by a further four cases with no proctoring.

Operative technique

All procedures were performed following a thorough consent process using the da Vinci Xi robotic system (Intuitive Surgical, Inc., Sunnyvale, California). Patients were positioned in steep Trendelenburg with a six-port transperitoneal approach. Pelvic lymph node dissection was performed in all cases to at least the level of the common iliac bifurcation. Urinary diversion was intracorporeal in all patients, with ileal conduit formation as the standard reconstruction. Uretero-enteric anastomoses were fashioned using either Wallace or Bricker techniques depending on intraoperative factors.

Perioperative care

All patients were enrolled in an Enhanced Recovery After Surgery (ERAS) programme. This included optimisation prior to surgery, early mobilisation, and early feeding to optimise patient recovery. Preoperative stoma siting was undertaken by a clinical nurse specialist. Thromboprophylaxis and antibiotic prophylaxis were administered according to institutional protocols. Postoperatively, patients received multimodal analgesia, early mobilisation, and early enteral nutrition.

Data collection

Demographic variables included age, sex, BMI, American Society of Anesthesiologists (ASA) grade, smoking history, and comorbidities. Operative data included operative time, blood loss, transfusion requirement, and conversion to open surgery. Pathological outcomes included tumour stage, nodal status, surgical margin status, and presence of variant histology. Complications were recorded and graded according to the Clavien-Dindo classification. Follow-up data included recurrence status, renal function, and postoperative readmissions or mortality.

Ethical approval was not required for this service evaluation. Data were anonymised, and analysis was performed using descriptive statistics compiled using Excel (Microsoft Corporation, Redmond, Washington).

## Results

**Figure 1:**
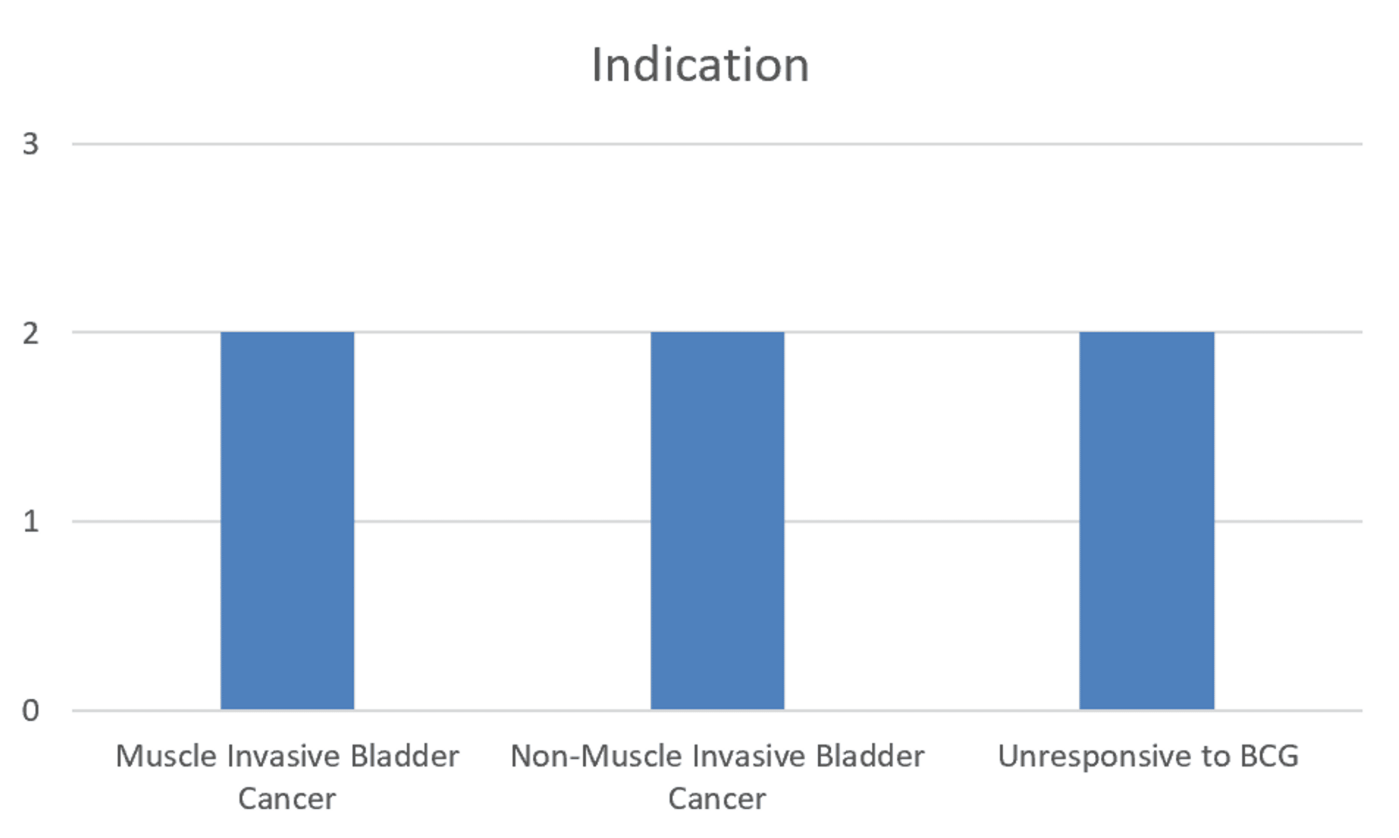
Bar chart indicating indication for cystectomy and respective number of patients for each indication

**Figure 2:**
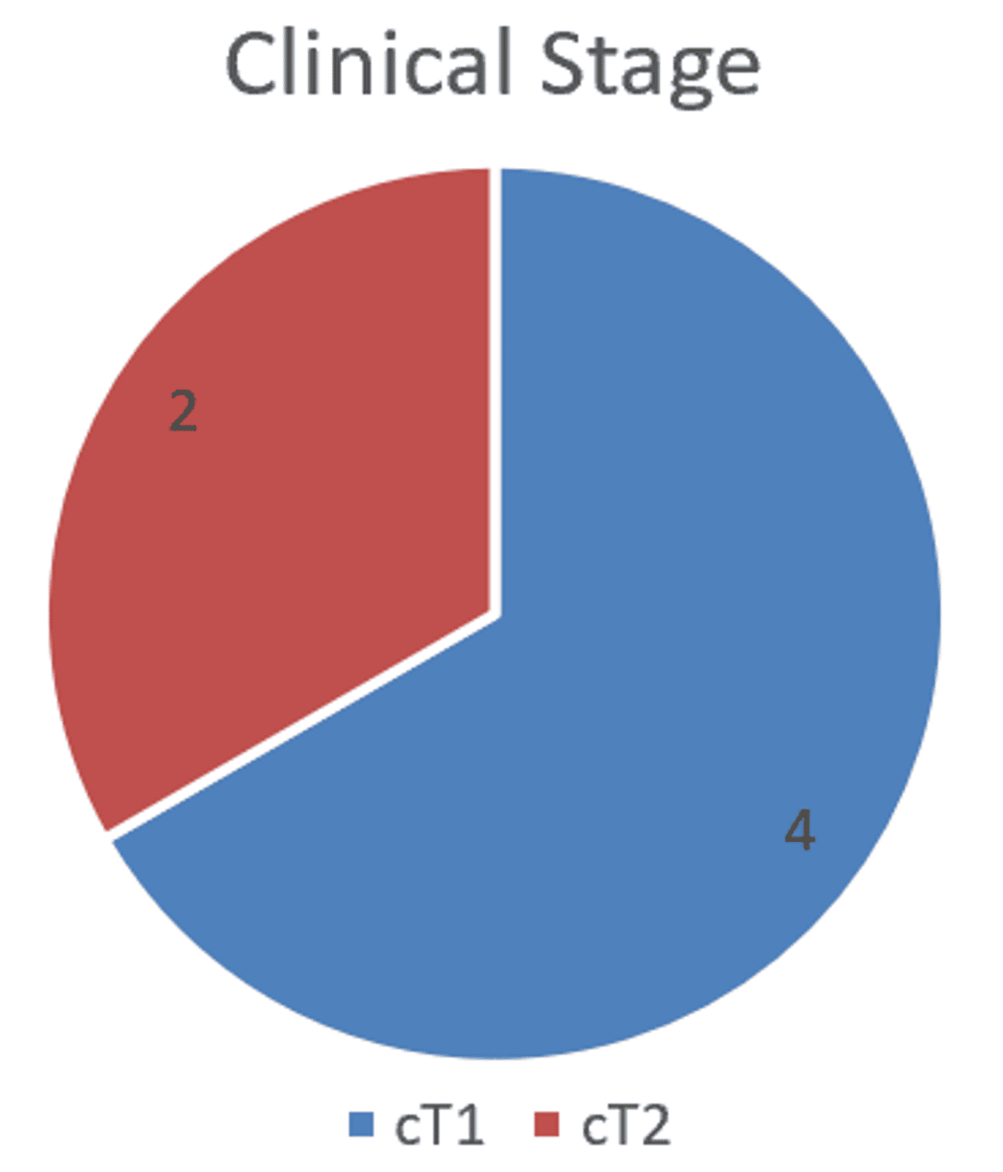
Pie chart indicating clinical staging for cases pre-operatively

A total of six patients underwent RARC during the study period. Indications for cystectomy included muscle-invasive bladder cancer in two patients, high-risk non-muscle-invasive bladder cancer in two patients, and two patients who had failed BCG treatment for bladder cancer (Figure 1). The median age was 65.5 years (range: 54-70 years), with four males and two females. The majority of patients were ASA grade II, and the median BMI was 29.1 kg/m². Three patients were ex-smokers, and comorbidities were limited, with one patient having mental health comorbidities and another a history of melanoma. Clinical staging preoperatively included four patients with cT1 disease and two patients with cT2 disease (Figure 2).

Operative metrics

**Figure 3:**
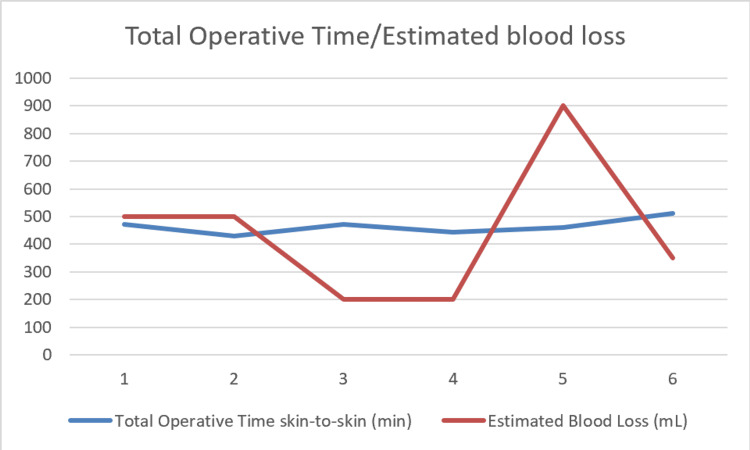
Line graph showing total operative time in minutes (blue) and estimated blood loss in millilitres (red)

The median total operative time was 466.5 minutes (range: 429-511 minutes) (Figure 3). The median estimated blood loss was 425 mL (range: 200-900 mL) (Figure 3), and no patients required intraoperative transfusion. All procedures were completed robotically with no conversions to open surgery. Urinary diversion was an intracorporeal ileal conduit in all patients.

Postoperative course

**Figure 4:**
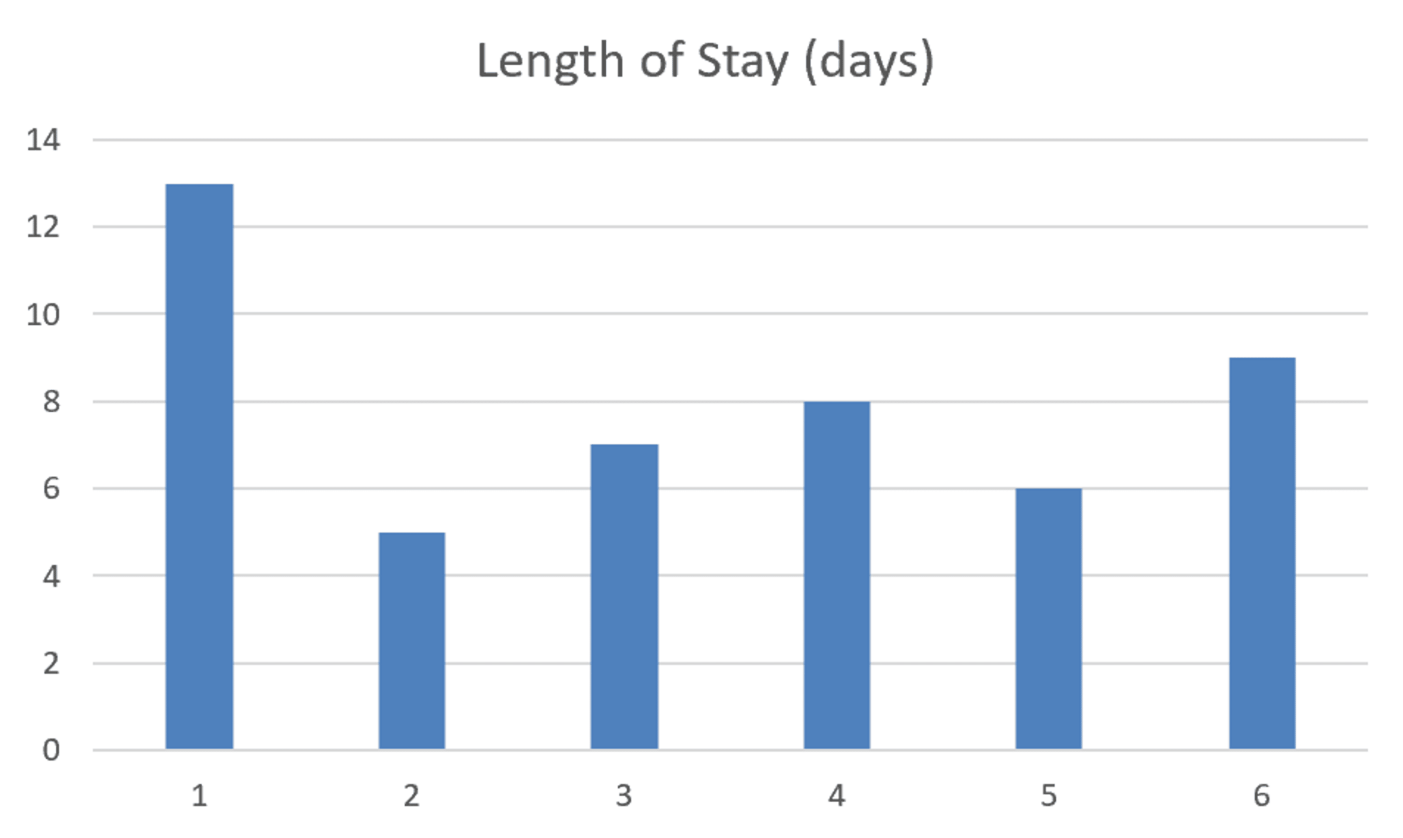
Bar chart showing length of stay in days

The median length of stay was 7.5 days (range: 5-13 days) (Figure 4). Two patients developed postoperative ileus requiring conservative management, and one patient developed stoma retraction, which was also managed conservatively. There were no returns to theatre and no 30-day readmissions. There was no 30-day or 90-day mortality.

Pathology

Pathological stages ranged from pTa to pT2a. Two patients had variant histology: one with nested urothelial carcinoma and one with small cell neuroendocrine carcinoma. Margins were negative in five patients, with one final pathology result pending at the time of reporting. Lymph node dissections yielded between five and 17 nodes, all of which were negative for malignancy.

Follow-up

The median follow-up period was short, with the longest follow-up being four months after surgery. All patients were well and disease-free at the last review, with preserved renal function and no major diversion-related complications.

## Discussion

This early case series demonstrates the safe and feasible introduction of RARC into a UK tertiary centre. Our outcomes are consistent with international reports demonstrating low perioperative morbidity, acceptable operative times, and negative margin rates [7, 10].

Comparisons with the literature

The RAZOR trial demonstrated the non-inferiority of RARC compared with ORC with respect to two-year progression-free survival (72.3% vs 71.6%) [10]. Our series, albeit limited by short follow-up, supports the oncological safety of RARC, with all patients disease-free at the last follow-up. Perioperative outcomes such as blood loss and length of stay are also comparable with published meta-analyses [8, 9].

Learning curve

Operative times in our series were longer than those typically reported from high-volume centres, reflecting the learning curve associated with introducing RARC [14, 15]. Studies suggest proficiency may be achieved after 30 to 50 cases, with continued improvement beyond 100 cases [14]. Our data demonstrate safe outcomes despite early positioning in this learning curve, highlighting the importance of structured training and proctorship.

Complications

Our cohort experienced minimal morbidity, with two cases of ileus and one stoma-related issue, all managed conservatively. This aligns with previous findings that while RARC does not eliminate complications, it may reduce the severity of perioperative events [7, 9]. The absence of major complications or readmissions within 30 days is reassuring, although larger series are required for validation.

Variant histology

The presence of a urothelial carcinoma with a large nested pattern and small cell neuroendocrine carcinoma in two of our patients underscores the importance of careful pathological review. Outcomes in these subtypes are less well defined, and their inclusion highlights the heterogeneity of real-world cystectomy populations. During the follow-up period of this review, the outcomes for these patients were the same as for the other four cases.

Implications for the NHS

The adoption of robotic surgery within the NHS has expanded rapidly, with over 80 robotic platforms in use nationwide [13]. While concerns regarding cost remain, studies suggest potential savings from reduced transfusions, complications, and hospital stays [16]. Our findings support the view that the safe adoption of robotic technology is achievable within publicly funded health systems when supported by multidisciplinary pathways [17-19].

Future directions

Future research should focus on long-term oncological outcomes, quality-of-life measures, and cost-effectiveness analyses. Particular attention should be paid to functional outcomes such as urinary and sexual function, which remain under-reported in the robotic cystectomy literature.

Limitations

This series is limited by the small sample size, short follow-up (the longest follow-up being four months), and retrospective design. Nevertheless, it provides important real-world data on the early implementation of RARC in a UK setting. Ongoing prospective data collection and inclusion in national registries will be essential to benchmark outcomes against international standards.

## Conclusions

Robotic-assisted radical cystectomy was safely introduced at our centre, with perioperative and pathological outcomes comparable to published international series. Despite the learning curve, no conversions or major complications occurred. These findings support the feasibility of RARC adoption within the NHS and indicate that it can be implemented safely, provided cases are selected appropriately and embedded within structured training programmes. Further research is required to establish long-term oncological outcomes, functional recovery, and cost-effectiveness.

## References

[1] Sung H, Ferlay J, Siegel RL, Laversanne M, Soerjomataram I, Jemal A, Bray F (2021). Global cancer statistics 2020: GLOBOCAN estimates of incidence and mortality worldwide for 36 cancers in 185 countries. CA Cancer J Clin.

[2] Cancer Research UK. Bladder cancer statistics. http://www.cancerresearchuk.org/health-professional/cancer-statistics/statistics-by-cancer-type/bladder-cancer.

[3] Martini A, Sfakianos JP, Renström-Koskela L (2020). The natural history of untreated muscle-invasive bladder cancer. BJU Int.

[4] Kiss B, Burkhard FC, Thalmann GN (2016). Open radical cystectomy: still the gold standard for muscle invasive bladder cancer. World J Urol.

[5] Gontero P, Birtle A, Capoun O (2024). European Association of Urology guidelines on non-muscle-invasive bladder cancer (TAT1 and carcinoma in situ)-a summary of the 2024 guidelines update. Eur Urol.

[6] Shabsigh A, Korets R, Vora KC (2009). Defining early morbidity of radical cystectomy for patients with bladder cancer using a standardized reporting methodology. Eur Urol.

[7] Nazzani S, Mazzone E, Preisser F (2018). Comparison of perioperative outcomes between open and robotic radical cystectomy: a population-based analysis. J Endourol.

[8] Tan WS, Lamb BW, Kelly JD (2015). Complications of radical cystectomy and orthotopic reconstruction. Adv Urol.

[9] Parekh DJ, Reis IM, Castle EP (2018). Robot-assisted radical cystectomy versus open radical cystectomy in patients with bladder cancer (RAZOR): an open-label, randomised, phase 3, non-inferiority trial. Lancet.

[10] NICE. Bladder cancer: diagnosis and management. NICE guideline NG2. http://www.ncbi.nlm.nih.gov/books/NBK551803/.

[11] Alfred Witjes J, Max Bruins H, Carrión A (2024). European Association of Urology guidelines on muscle-invasive and metastatic bladder cancer: summary of the 2023 guidelines. Eur Urol.

[12] Maynou L, McGuire A, Serra-Sastre V (2024). Efficiency and productivity gains of robotic surgery: the case of the English National Health Service. Health Econ.

[13] Hayn MH, Hellenthal NJ, Seixas-Mikelus SA, Mansour AM, Stegemann A, Hussain A, Guru KA (2011). Is patient outcome compromised during the initial experience with robot-assisted radical cystectomy? Results of 164 consecutive cases. BJU Int.

[14] Khan MS, Elhage O, Challacombe B, Rimington P, Murphy D, Dasgupta P (2011). Analysis of early complications of robotic-assisted radical cystectomy using a standardized reporting system. Urology.

[15] Leow JJ, Reese SW, Jiang W (2014). Propensity-matched comparison of morbidity and costs of open and robot-assisted radical cystectomies: a contemporary population-based analysis in the United States. Eur Urol.

[16] Aminoltejari K, Hird AE, Klaassen Z (2023). Robotic versus open cystectomy for bladder cancer: Synthesizing the data from current systematic reviews and meta-analyses. Ann Surg Oncol.

[17] Yuh B, Wilson T, Bochner B (2015). Systematic review and cumulative analysis of oncologic and functional outcomes after robot-assisted radical cystectomy. Eur Urol.

[18] Moschovas MC, Manzano JP, Patel V (2023). Robotic intracorporeal urinary diversion: principles, applications, and future directions. Robotic Surgery Devices in Urology.

[19] Challacombe BJ, Bochner BH, Dasgupta P (2011). The role of laparoscopic and robotic cystectomy in the management of muscle-invasive bladder cancer with special emphasis on cancer control and complications. Eur Urol.

